# Periconceptional ethanol exposure induces a sex specific diuresis and increase in AQP2 and AVPR2 in the kidneys of aged rat offspring

**DOI:** 10.14814/phy2.14273

**Published:** 2019-11-06

**Authors:** Emily S. Dorey, Sarah L. Walton, Jacinta I. Kalisch‐Smith, Tamara M. Paravicini, Emelie M. Gardebjer, Kristy A. Weir, Reetu R. Singh, Helle Bielefeldt‐Ohmann, Stephen T. Anderson, Mary E. Wlodek, Karen M. Moritz

**Affiliations:** ^1^ School of Biomedical Sciences The University of Queensland Brisbane Queensland; ^2^ School of Veterinary Science The University of Queensland Brisbane Queensland; ^3^ The Department of Physiology The University of Melbourne Melbourne Victoria; ^4^ Child Health Research Centre The University of Queensland Brisbane Queensland

**Keywords:** Alcohol, aquaporin‐2, kidney, renal function

## Abstract

Maternal alcohol consumption can impair renal development and program kidney dysfunction in offspring. Given that most women who drink alcohol cease consumption upon pregnancy recognition, we aimed to investigate the effect of alcohol around the time of conception (PC:EtOH) on offspring renal development and function. Rats received a liquid diet ±12.5% v/v ethanol from 4 days before to 4 days after mating. At postnatal day 30, nephron number was assessed. Urine flow and electrolyte (Na, K, Cl) excretion was measured at 6 and 19 months and blood pressure at 12 months. At 19 months, kidneys were collected for gene and protein analysis and assessment of collecting duct length. At postnatal day 30, PC:EtOH offspring had fewer nephrons. At 6 months, PC:EtOH exposure did not alter urine flow nor affect blood pressure at 12 months. At 19 months, female but not male offspring exposed to PC:EtOH drank more water and had a higher urine flow despite no differences in plasma arginine vasopressin (AVP) concentrations. *Aqp2* mRNA and *Avpr2* mRNA and protein expression was increased in kidneys from female PC:EtOH offspring but collecting duct lengths were similar. Immunofluorescent staining revealed diffuse cytoplasmic distribution of AQP2 protein in kidneys from PC:EtOH females, compared with controls with apical AQP2 localization. PC:EtOH resulted in a low nephron endowment and in female offspring, associated with age‐related diuresis. Changes in expression and cellular localization of AQP2 likely underpin this disturbance in water homeostasis and highlight the need for alcohol to be avoided in early pregnancy.

## Introduction

The incidence of alcohol consumption during pregnancy is high in western countries, with approximately 30% of surveyed women in the United States reporting drinking at some point during their pregnancy compared with 40% of Australian women and up to 80% of women in Ireland (Ethen et al. 2009; O'Keeffe et al. 2015). While many women stop or at least reduce their alcohol intake once they are aware of their pregnancy, research shows that approximately half of pregnancies in the United States are unintended and therefore, it is likely that many women are exposing their embryos to alcohol prior to pregnancy recognition (Finer and Zolna, 2014). Alcohol is known to impair the development of the central nervous system and have long lasting effects on offspring behavioral outcomes (Riley et al. 2011). Whilst most studies have focused on the developing brain, less is known about the effects of maternal alcohol consumption on the development of other organs, such as the kidney. The impact of prenatal ethanol exposure on the developing kidney is important given models of uteroplacental insufficiency, maternal low protein consumption, and fetal hypoxia have all been shown to impair renal development, reduce nephron number, and program renal and/or cardiovascular disease in offspring (for review see (Dorey et al. 2014)). Importantly, many of these studies have shown sex differences in both the immediate effects on the developing kidney, and the subsequent development of renal and cardiovascular disease in adulthood.

We recently undertook a systematic review of the literature to identify studies in which renal development and/or function had been examined following prenatal exposure (Reid et al. 2019). This identified a small number of studies that suggested prenatal alcohol exposure could impair kidney development and cause alterations in renal electrolyte excretion. Alcohol exposure during late gestation also reduced nephron number in fetal sheep (Gray et al. 2008). Additionally, in rats exposed to high levels of alcohol for 2 days during mid‐gestation, nephrogenesis was impaired and offspring developed sex‐specific changes in glomerular filtration rate and protein excretion at 6 months of age (Gray et al. 2010). In adulthood, these rats also developed higher mean arterial pressure compared to same sex controls. This correlation between decreased nephron number and increased blood pressure, known as the ‘Brenner hypothesis’ (Brenner et al. 1988), has been implicated following many prenatal perturbations including low protein diet during the periconceptional period (Watkins et al. 2008) and dexamethasone in mid gestation (Singh et al. 2007). Exposure to high levels of alcohol during late gestation in the rat resulted in renal dysfunction in adulthood, with increased urine flow and water consumption observed, particularly in female offspring (Knee et al. 2004). This increased urine flow was associated with a decrease in arginine vasopressin (AVP) content in the pituitary and decreased *Avp* mRNA expression in the hypothalamus. Furthermore, the AVP response to hemorrhage was reduced in ethanol‐exposed offspring indicating an inability to respond to rapid hypovolemia (Knee et al. 2004; Bird et al. 2006). High levels of alcohol during pregnancy in the rat are known to result in elevated levels of circulating AVP in association with increased water consumption in the offspring (Dow‐Edwards et al. 1989). Additionally, ethanol ingestion spanning pre‐pregnancy and pregnancy increases renal aquaporin (AQP) 2 and AQP3 protein expression in both the mothers and their offspring (Garcia‐Delgado et al. 2004) suggesting that prenatal alcohol exposure can affect central control of volume homeostasis via AVP secretion as well as renal mechanisms of urine concentrating ability.

The majority of studies have examined the effects of maternal perturbations either during the entirety of pregnancy or during mid/late gestation. More recently, studies have demonstrated that perturbations around the time of conception result in long lasting impairments of offspring physiology (McMillen et al. 2008). Given most women abstain or reduce alcohol consumption upon confirmation of pregnancy, the current study sought to investigate the impact of periconceptional alcohol consumption on fetal kidney development and offspring physiology. We have previously reported that alcohol consumption in the rat during the periconceptional period induces fetal growth restriction and altered placental morphology (Gardebjer et al. 2014), resulting in adult offspring with insulin resistance (Gardebjer et al. 2015) and males were more likely to become obese (Gardebjer et al. 2018). Furthermore, female offspring were found to consume more water during an ethanol preference test following periconceptional alcohol exposure (Dorey et al. 2018). In this study, we hypothesized that periconceptional alcohol exposure would decrease nephron endowment, culminating in functional deficits in the adult kidney and elevated blood pressure. Furthermore, we suggest that the renal deficits would be more evident in female offspring and worsen with age.

## Materials and Methods

### Ethical approval

All experiments were performed at the University of Queensland in accordance with the ethical standards of *The Australian Code for the Care and Use of Animals for Scientific Purposes* and were approved by the University of Queensland Anatomical Bioscience Animal Ethics Committee before commencement of the study.

### Animal treatment and offspring measures

Outbred female Sprague Dawley rats were housed individually on a 12 h light/dark cycle with the dark period commencing at 12 pm. Female rats in estrous (designated embryonic day −4, E‐4) were randomly assigned to either a liquid diet containing 12.5% v/v ethanol (PC:EtOH, *n* = 12) or a control diet (Control, *n* = 12), as described previously (Gardebjer et al. 2015). The complete liquid diet consisted of Sustagen hospital formula (Mead Johnson, Auckland, New Zealand), corn flour, Selenium (Selemite B; Blackmores, Warriewood, NSW, Australia), low fat milk, sunflower oil, 50 mmol/L Copper (II) sulfate (Sigma Aldrich, Sydney, NSW, Australia), 199 mmol/L Ferric citrate (Sigma Aldrich), 303 mmol/L manganese sulfate (Sigma Aldrich). As previously reported, the 12.5% ethanol diet was modified to give equal energy percentages of protein, fat and calories based on consumption when compared to control diet (Gardebjer et al. 2014). The diet was offered ad libitum fresh each day at 12 pm (the commencement of the dark cycle) and the volume of diet consumed recorded at 5 pm and at 9 am the following morning. At this time, the diet was removed and animals were offered water for 3 h. When entering estrous for a second time (E0), they were placed for 5 h with a male. Presence of seminal plugs indicated successful mating. The liquid diet was continued until E5 when dams were returned to standard chow (meat free rat and mouse chow, Specialty Feeds) for the remainder of pregnancy and lactation. At day 21, offspring were weaned onto this same diet.

A subset of offspring were euthanized at postnatal day 30 (PN30, one male and one female from each litter, PC:EtOH *n* = 11, Control *n* = 9) using 50:50 ketamine and xylazine (0.1 mL/kg body weight, Lyppard Australia Ltd., Australia) and kidneys were collected and weighed. The right kidneys were immersion fixed in 4% paraformaldehyde (PFA) for determination of glomerular (nephron) number and collecting duct lengths. In a second subset of dams (PC:EtOH *n* = 12 and Control *n* = 12), offspring were aged for physiological studies.

At 6 months of age, blood (1 mL) was collected in EDTA from a subset of non‐fasted offspring via tail tip and plasma frozen at −80°C for later analysis of AVP followed by renal function measurements (as described below). In a subset of animals, blood pressure was measured by radiotelemetry at 12 months, whilst a separate set of animals was aged until 19 months when renal function was re‐assessed under basal conditions and following 24 h dehydration. Following these measures, rats were fasted overnight before being euthanized with 0.1 mL/kg of sodium pentobarbital. Blood was collected via cardiac puncture, placed in EDTA coated collection tubes and centrifuged at 4°C for 10 min at 900 *g* and stored at −80°C. Kidneys were collected and fixed in 4% PFA for histology or frozen at −80°C for molecular analyses.

### Renal function

At 6 and 19 months of age, rats were acclimatized to individual metabolic cages in the days prior to the experimental urine collection. Rats were then placed in the metabolic cages for 24 h, with food and water consumption and urine output recorded before being normalized for body weight. A sample of urine was collected and frozen at −20°C for later analysis of urinary Na^+^, K^+^, Cl^−^ (6 and 19 months) and urinary albumin and creatinine (19 months). After a 24 h recovery period, rats were placed in metabolic cages for a period of 24 h without access to water to assess the response to a water deprivation challenge. Urine output was recorded before being normalized to body weight and urine was collected and stored at −20°C. Following the dehydration challenge, a tail vein blood sample was collected, treated with EDTA, plasma collected and stored at −80°C for analysis of AVP concentrations.

### Blood pressure

Blood pressure was analysed at 12 months in freely moving unrestrained rats using radiotelemetry (model PA‐C40; Data Sciences International, MN). Briefly, following induction of anaesthesia, rats were maintained under anesthesia using 2% isoflurane in oxygen. The femoral artery was located and cleaned before the radiotelemetry cannula was inserted. Animals were allowed to recover for 10 days prior to measurements being recorded. At the end of the recovery period, measurements of systolic blood pressure (SBP), diastolic blood pressure (DBP), pulse pressure (PP), mean arterial pressure (MAP) and heart rate (HR) were acquired for 10 sec every 15 min for 3 days.

### Urinary and plasma analyses

Urine and plasma electrolytes were analysed using the Cobas Integra 400 Chemistry Analyzer (Block Scientific, NY). Osmolality was determined using a *µ*OSMETTE freeze point osmometer (Precision Systems, MA). Urinary albumin, creatinine, and cyclic AMP (cAMP) were evaluated using commercially available kits (Nephrat (Exocell, PA); Creatinine Companion (Exocell, PA); Cyclic AMP Select ELISA Kit (Cayman chemical, MI) respectively. Plasma AVP levels were determined using Vasopressin Direct RIA (Buhlmann Laboratories, Switzerland). Urinary electrolytes and cAMP were normalized for flow normalized to body weight.

### Glomerular number, collecting duct lengths, and renal pathology

Glomerular number was estimated in kidneys of male and female offspring at PN30 (*n* = 8 per treatment per sex). Briefly, fixed kidneys were processed to paraffin and the entire kidney exhaustively sectioned at 5 *µ*m (pole‐to‐pole). Ten evenly spaced section pairs were systematically sampled and stained with the lectin peanut agglutinin (*Arachis hypogea*, PNA; Sigma Aldrich, Castle Hill, NSW, Australia) and the physical disector/fractionator combination was used to count the number of glomeruli from each section pair as previously described (Cullen‐McEwen et al. 2011).

Histopathology was assessed in paraffin‐embedded kidney sections at 19 months. Representative 4 *µ*m midline sections were stained with periodic acid‐Schiff stain (PAS) for pathological characterization (*n* = 3 per treatment). Sections were assessed by an expert pathologist blinded to treatment groups.

Total collecting duct length within kidneys from female offspring at PN30 and 19 months of age was estimated using our recently‐described design‐based stereological method (Walton et al. 2016). In brief, kidneys were processed to paraffin and exhaustively sectioned at 5 *μ*m. Ten evenly spaced sections were systematically sampled across the length of each kidney. All sections were immunohistochemically labeled with AQP2 to mark the collecting ducts (1:1000, AB3274, Millipore) as previously described (Walton et al. 2016). Total collecting duct length was estimated using the cycloid arc test system within the StereoInvestigator software package (MBF Bioscience, Williston, VT) (Walton et al. 2016).

### Real time PCR and Western blotting

RNA was extracted from kidneys using the RNeasy Mini Kit (Qiagen, VIC, Australia). All samples were DNase1 treated. RNA was reverse transcribed into cDNA using iScript (Bio‐Rad, NSW, Australia). qPCR was performed using custom made *Aqp2* primers and probes (Fwd: CCCTCTCCATTGGTTTCTCTGTTAC, Rev: AGGGAGCGGGCTGGATTC, Probe: CTGGGCCACCTCCTTGGGATCTA; Biosearch Tech) and assays on demand for *Avpr2* (Rn00569508_g1) using Taqman reagents (Applied Biosystems, CA). Results were analysed using the ∆∆Ct method and 18s used as a housekeeping target.

For AVPR2 protein analysis, kidney samples were homogenized (2 × 20 sec) in RIPA buffer prior to centrifugation (3000 *g*, 20 min, 4°C). For AQP2 protein analysis, samples were homogenized (2 × 20 sec) using a sucrose isolation buffer (250 mmol/L sucrose, 10 mmol/L triethanolamine, pH 7.6, adapted from (Cano‐Penalver et al. 2014; Tian et al. 2004). Protein concentration was established using the RC DC™ Bio‐Rad protein assay. Twenty *µ*g (5–7 per group) of protein was subjected to SDS‐page (12% polyacrylamide gel), transferred to a low florescence Polyvinylidene fluoride (PVDF) membrane and blocked in 4% fish gelatin with goat serum (1:10). Primary antibody (AVPR2: AVP Receptor V2 (H‐80), Santa Cruz Biotechnology, Dallas, Texas; AQP2: Rabbit Anti‐Aquaporin 2 (AB3274‐200 *µ*L, EMD Millipore Corporation, CA); Anti‐Aquaporin2 Phospho S256 (ab109926, Abcam, Cambridge, UK) was applied overnight at 4°C. Beta Actin (BACT) applied for an hour at room temperature and secondary antibodies (LI‐COR IRDye 680 goat anti‐rabbit and IRDye 800CW goat anti‐mouse, Millennium Science, Mulgrave, Australia) were used to quantify protein expression using LI‐COR Odyssey infrared imaging system (LI‐COR Biosciences, Lincoln, NE).

### Renal AQP2 localization

APQ2 localization was performed at 19 months using immunofluorescence staining. Representative midline kidney sections were dewaxed, and antigen retrieval was performed in 10 mmol/L citrate buffer (pH 8.5) for 20 min at 80°C. Sections were blocked in 10% goat serum in 2% BSA for 30 min at room temperature. AQP2 antibody (AB3274, Merck Group, Bayswater, 1:500) was applied and sections incubated overnight at 4°C. Following washes, sections were incubated with AlexaFluor 488 goat anti rabbit IgG for 1 h and counterstained with Hoechst to detect nuclei. Images were collected using a Leica DMi8 Inverted Confocal microscope (representative pictures of cortex and medulla, 1 per animal, *n* = 3).

### Statistics

Data are presented as mean ± standard error of the mean (SEM). For renal function, nephron number, organ weight, urinary and plasma electrolytes and gene expression, a two‐way ANOVA with treatment (PTrt) and sex (PSex) as factors, and Bonferroni post‐hoc analyses were used where appropriate. Protein expression and collecting duct length data were analysed using Student's *t*‐tests. Blood pressure results were analysed using a two‐way repeated measures ANOVA with Bonferroni post‐hoc analysis. *P* < 0.05 was considered statistically significant.

## Results

### Maternal dietary intake

As reported previously, animals on the alcohol diet have a similar caloric intake to control animals both during the periconceptional exposure and the remainder of pregnancy (Gardebjer et al. 2014). Dams on the alcohol diet consumed more water during the periconceptional period (6–7 mL/day compared to 3–4 mL/day in control animals (Gardebjer et al. 2014). Offspring in both groups consumed similar amounts of food (Dorey et al. 2018).

### Organ weights and kidney stereology at PN30

**Figure 1:**
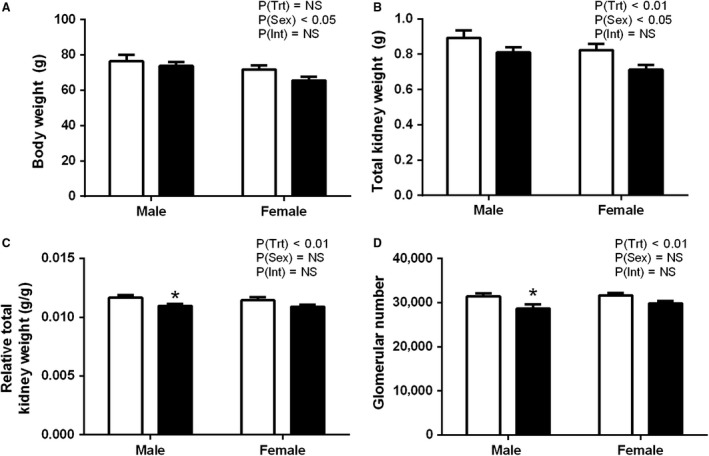
Body weight (A), total kidney weight (B), total kidney weight relative to body weight (C), and estimated glomerular number (D) in offspring at PN30 from dams that consumed a control (white) or PC:EtOH diet (black). Data are mean ± SEM. *n* = 8–10 per group. Data were analysed using two‐way ANOVA with Bonferroni post‐hoc analysis. *P < 0.05 when compared with control of the same sex. NS, not significant.

At PN30, PC:EtOH did not alter body weight but did result in a decrease in total kidney weight (PTrt < 0.01, Fig. 1A and 1B). PC:EtOH similarly decreased total kidney to body weight ratio (PTrt < 0.01, Fig. 1C), with post‐hoc analysis demonstrating that this reduction was significant in male PC:EtOH offspring (*P* < 0.05). Females were lighter and had smaller kidneys than males (PSex < 0.05). Overall, PC:EtOH reduced glomerular number at PN30 (PTrt < 0.01, ~9% in males, ~6% in females, Fig. 1D) but post‐hoc analysis demonstrated that this was only significant in male PC:EtOH offspring (*P* < 0.05). Total collecting duct length did not differ between control and PC:EtOH females at PN30 (Control: 50 ± 4 m; PC:EtOH: 48 ± 2 m).

### Renal function at 6 months of age

**Figure 2:**
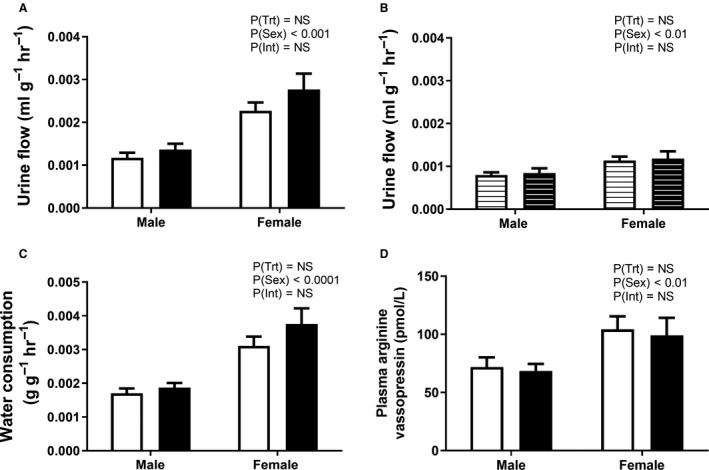
Urine flow under basal (A, solid bars) and dehydration (B, lined bars) conditions, water consumption (C) and plasma arginine vasopressin levels (D) in offspring at 6 months of age from dams that consumed a control (white) or PC:EtOH diet (black). Data are mean ± SEM. *n* = 5–10 per group. Data were analysed using two‐way ANOVA with Bonferroni post‐hoc analysis. NS, not significant.

At 6 months of age, PC:EtOH did not affect urine flow under basal or dehydrated conditions compared with controls (Figure 2A and 2). Basal plasma AVP concentrations were also similar between groups (Figure 2D). Under basal conditions PC:EtOH did not alter water intake in offspring, though females consumed more water than males regardless of treatment group (PSex < 0.05, Fig. 2C). Females had higher urine flow than males in both basal and dehydration conditions (PSex < 0.05, Fig. 2A and 2) and higher basal concentrations of plasma AVP (PSex < 0.05, Fig. 2D).

### Blood pressure and heart rate

**Figure 3:**
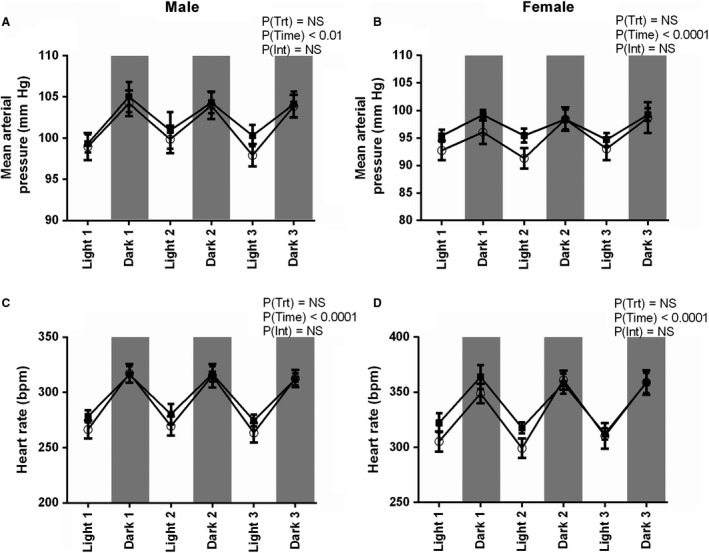
Mean arterial pressure (A,B) and heart rate (C,D) over 3 day light and dark in male (A,C) and female (B,D) offspring at 12 months from dams that consumed control (white circles) or PC:EtOH (black squares) diet. Data are mean ± SEM. *n* = 7–9. Data were analysed using a two‐way repeated measures ANOVA with Bonferroni post‐hoc analysis. NS, not significant.

At 12 months of age PC:EtOH did not alter SBP, DBP, MAP, or PP in either sex (Table 1, Fig. 3A and 3). In both males and females there was an effect of time of day (PTime < 0.05) for all variables measured, with increases during the dark cycle. There was a significant interaction between time of day and the three day average heart rate in male PC:EtOH offspring (HR, PInt < 0.05), but post‐hoc analysis did not show any significant differences (Table 1).

**Table 1:** Blood pressure parameters as measured by radiotelemetry averaged over three light and dark cycles.

	PC treatment	Control	PC:EtOH	Control	PC:EtOH	Statistics
	Light cycle	Light	Light	Dark	Dark	
Male	SBP (mmHg)	124 ± 1	126 ± 1	129 ± 1	130 ± 2	P(Trt) = NS
						P(Time) < 0.0001
						P(Int) = NS
	DBP (mmHg)	82 ± 1	82 ± 1	87 ± 1	86 ± 1	P(Trt) = NS
						P(Time) < 0.0001
						P(Int) = NS
	PP (mmHg)	42 ± 1	44 ± 1	43 ± 1	45 ± 1	P(Trt) = NS
						P(Time) < 0.01
						P(Int) = NS
	HR (bpm)	266 ± 8	278 ± 6	314 ± 9	315 ± 5	P(Trt) = NS
						P(Time) < 0.0001
						P(Int) < 0.05
Female	SBP (mmHg)	116 ± 2	118 ± 1	123 ± 2	122 ± 1	P(Trt) = NS
						P(Time) < 0.01
						P(Int) = NS
	DBP (mmHg)	74 ± 2	77 ± 1	79 ± 2	81 ± 1	P(Trt) = NS
						P(Time) < 0.05
						P(Int) = NS
	PP (mmHg)	43 ± 1	41 ± 1	43 ± 1	41 ± 1	P(Trt) = NS
						P(Time) = NS
						P(Int) = NS
	Heart rate (bpm)	305 ± 9	317 ± 5	356 ± 9	360 ± 10	P(Trt) = NS
						P(Time) < 0.0001
						P(Int) = NS

Data are mean ± SEM and analysed by two‐way ANOVA. Systolic blood pressure (SBP), diastolic blood pressure (DBP), pulse pressure (PP), and heart rate (HR) averaged over three consecutive days and nights. *n* = 7–9. NS = not significant.

### Renal function in aged animals

**Figure 4:**
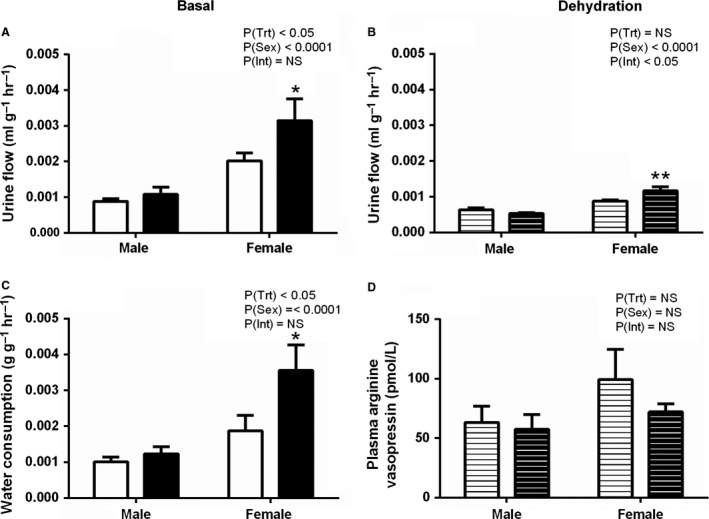
Urine flow (A) and water consumption (C) under basal conditions (solid bars) and urine flow (B) and plasma arginine vasopressin levels during dehydration (D) comparing offspring from dams that consumed control (white) or PC:EtOH (black) diet at 19 months of age. Data are mean ± SEM. *n* = 6–9. Data were analysed using two‐way ANOVA with Bonferroni post‐hoc analysis. *P < 0.05 and **P < 0.01 when compared to same sex control. NS, not significant.

At 19 months, females had greater urine flow than males under both basal and dehydration conditions (PSex < 0.0001, Fig. 4A and 4B). PC:EtOH treatment increased basal urine flow (PTrt < 0.05, Fig. 4A), with post‐hoc analysis demonstrating female offspring in the PC:EtOH group had significantly higher flow than that of control female offspring (*P* < 0.05). PC:EtOH increased water consumption (PTrt < 0.05, Fig. 4C) with post‐ho*c* analysis indicating female PC:EtOH offspring consumed more water per hour than control females (*P* < 0.05). Additionally, female offspring consumed more water than males (PSex < 0.01) regardless of treatment. There was a significant interaction between treatment and sex when assessing urine flow during dehydration (PInt < 0.01, Fig. 4B). Post‐hoc analysis showed that female PC:EtOH offspring had significantly higher urine flow than control female offspring (*P* < 0.01). Plasma AVP concentrations following dehydration were not affected by the PC:EtOH treatment in either sex (Fig. 4D).

### Urine and plasma analysis at 19 months

At 19 months of age under basal conditions, PC:EtOH had no effect on electrolyte excretion, urinary excretion of creatinine or albumin, the urinary albumin:creatinine ratio, urine osmolality or urinary cAMP excretion (Table 2). At 19 months, females had higher rates of U_Na_V, U_Cl_V, and U_K_V compared to males (PSex < 0.0001). Females of both treatment groups had a greater urinary albumin:creatinine ratio (PSex < 0.05), lower urine osmolality (PSex < 0.01), lower levels of urinary creatinine (PSex < 0.01), and higher urinary albumin (PSex < 0.05) concertation than males. Plasma sodium, potassium, and chloride concentrations were not different between treatment groups (Table 3).

**Table 2:** Urinary analysis, and body and kidney weights at 19 months of age.

			Male		Female		Statistics
			Control	PC:EtOH	Control	PC:EtOH	
Urine	Basal	U_Na_V (*µ*mol/g/24 h)	1.29 ± 0.20	1.25 ± 0.18	2.03 ± 0.27	2.80 ± 0.28	P(Trt) = NS
							P(Sex) < 0.0001
							P(Int) = NS
		U_K_V (*µ*mol/g/24 h)	3.12 ± 0.31	3.07 ± 0.30	4.45 ± 0.26	5.26 ± 0.46	P(Trt) = NS
							P(Sex) < 0.0001
							P(Int) = NS
		U_Cl_V (*µ*mol/g/24 h)	2.22 ± 0.27	2.07 ± 0.24	3.68 ± 0.27	4.50 ± 0.48	P(Trt) = NS
							P(Sex) < 0.0001
							P(Int) = NS
		Creatinine (mg/dL)	126.6 ± 7.4	147.1 ± 14.9	80.0 ± 6.4	96.4 ± 16.1	P(Trt) = NS
							P(Sex) < 0.001
							P(Int) = NS
		Albumin (mg/dL)	20.59 ± 1.76	19.98 ± 3.79	28.06 ± 3.77	30.41 ± 3.96	P(Trt) = NS
							P(Sex) < 0.05
							P(Int) = NS
		Albumin: creatinine	0.18 ± 0.01	0.18 ± 0.70	0.40 ± 0.84	0.46 ± 0.17	P(Trt) = NS
							P(Sex) < 0.05
							P(Int = NS
		OSM (mOsm/kg H_2_0)	1155 ± 76	1090 ± 107	849.8 ± 61	692.3 ± 92	P(Trt) = NS
							P(Sex) < 0.001
							P(Int) = NS
		cAMP (pmol/mL/g/24 h)	12.60 ± 2.62	12.98 ± 1.78	6.71 ± 0.82	8.64 ± 0.79	P(Trt) = NS
							P(Sex) < 0.01
							P(Int) = NS
	Dehydration	U_Na_V (*µ*mol/g/24 h)	1.38 ± 0.17	1.20 ± 0.17	1.74 ± 0.08	1.67 ± 0.30	P(Trt) = NS
							P(Sex) < 0.05
							P(Int) = NS
		U_K_V (*µ*mol/g/24 h)	2.61 ± 0.18	2.49 ± 0.22	3.04 ± 0.24	3.56 ± 0.44	P(Trt) = NS
							P(Sex) < 0.01
							P(Int) = NS
		U_Cl_V (*µ*mol/g/24 h)	2.13 ± 0.19	2.00 ± 0.24	2.59 ± 0.16	2.61 ± 0.34	P(Trt) = NS
							P(Sex) < 0.05
							P(Int) = NS
		OSM (mOsm/kg H_2_0)	1712 ± 145	1861 ± 102	1429 ± 101	1231 ± 130	P(Trt) = NS
							P(Sex) < 0.001
							P(Int) = NS
	% Change	Urine flow (mL/g/24 h)	−24.5 ± 4.7	−30.6 ± 7.3	−44.5 ± 5.6	−54.2 ± 9.0	P(Trt) = NS
							P(Sex) < 0.01
							P(Int) = NS
		OSM (mOsm/kg H_2_0)	48.0 ± 6.4	80.7 ± 17.2	70.3 ± 9.5	95.0 ± 34.0	P(Trt) = NS
							P(Sex) = NS
							P(Int) = NS
Aged animal algometry		Body weight (g)	780 ± 46	767 ± 30	460 ± 17	471 ± 33	P(Trt) = NS
							P(Sex) < 0.0001
							P(Int) = NS
		Total kidney (g)	3.56 ± 0.31	3.25 ± 0.25	2.27 ± 0.05	2.49 ± 0.13	P(Trt) = NS
							P(Sex) < 0.0001
							P(Int) = NS
		Total kidney/BW (g/g)	0.0046 ± 0.0004	0.0050 ± 0.0060	0.0050 ± 0.0055	0.0054 ± 0.0062	P(Trt) = NS
							P(Sex) = NS
							P(Int) = NS

Data represented are mean ± SEM and *n* = 6–9 per group. Data analysed by **two‐way ANOVA** with Bonferroni post‐hoc test. NS = not significant.

In response to dehydration, urine flow decreased compared to baseline in all groups (Table 2). The decrease in urine flow in response to dehydration was similar between the control and PC:EtOH groups regardless of sex (Table 2). Female offspring reduced urine flow (% change) more than males under the dehydration challenge, irrespective of periconceptional exposure (PSex < 0.01; Table 2). PC:EtOH had no effect on urinary electrolytes or osmolality at 19 months during dehydration, though U_Na_V (*P* < 0.05), U_Cl_V (PSex < 0.05), and U_K_V (PSex < 0.05) remained higher in females compared to males. Under dehydration conditions, males had higher urine osmolality than females (PSex < 0.01; Table 2), though the % change in osmolality between basal and dehydration conditions was not different between sexes. PC:EtOH did not affect plasma osmolality, though female offspring had more concentrated plasma compared to males regardless of treatment group (PSex < 0.05, Table 3). There were no alterations of sodium, potassium and chloride levels in plasma obtained under basal and dehydration conditions following PC:EtOH.

**Table 3:** Analysis of plasma collected under basal and dehydration conditions at 19 months of age.

			Male		Female		Statistics
			Control	PC:EtOH	Control	PC:EtOH	
Plasma	Basal	Plasma Na^+^ (mmol/L)	131.7 ± 3.9	130.3 ± 4.6	135.8 ± 8.7	132.6 ± 2.1	P(Trt) = NS
							P(Sex) = NS
							P(Int) = NS
		Plasma K^+^ (mmol/L)	4.8 ± 0.2	4.9 ± 0.2	4.7 ± 0.2	4.6 ± 0.2	P(Trt) = NS
							P(Sex) = NS
							P(Int) = NS
		Plasma Cl^‐^ (mmol/L)	83.9 ± 2.6	83.0 ± 3.3	87.0 ± 4.9	82.4 ± 1.8	P(Trt) = NS
							P(Sex) = NS
							P(Int) = NS
		OSM (mOsm/kg H_2_0)	261.8 ± 8.2	258.3 ± 9.4	280.8 ± 3.0	276.0 ± 4.8	P(Trt) = NS
							P(Sex) < 0.05
							P(Int) = NS
	Dehydration	Plasma Na^+^ (mmol/L)	144.3 ± 8.2	146.2 ± 2.7	148.7 ± 2.1	150.4 ± 3.0	P(Trt) = NS
							P(Sex) = NS
							P(Int) = NS
		Plasma K^+^ (mmol/L)	5.5 ± 0.3	10.6 ± 4.4	5.7 ± 0.5	5.7 ± 1.0	P(Trt) = NS
							P(Sex) = NS
							P(Int) = NS
		Plasma Cl^‐^ (mmol/L)	93.7 ± 5.5	93.7 ± 2.4	94.4 ± 1.5	95.6 ± 2.2	P(Trt) = NS
							P(Sex) = NS
							P(Int) = NS
		OSM (mOsm/kg H_2_0)	284.1 ± 6.6	282.6 ± 6.2	298.7 ± 4.3	298.5 ± 3.1	P(Trt) = NS
							P(Sex)<0.01
							P(Int) = NS

Data represented are mean ± SEM and *n* = 6–9 per group. Data analysed by **two‐way ANOVA** with Bonferroni post‐hoc test. NS = not significant.

### Post‐mortem weights at 19 months

At 19 months of age, PC:EtOH had no effect on body weight, kidney weight or relative kidney weight of offspring (Table 2). Female offspring were lighter (PSex < 0.0001) and had smaller kidneys (PSex < 0.0001) than male offspring.

### Renal histology in aged offspring

**Figure 5:**
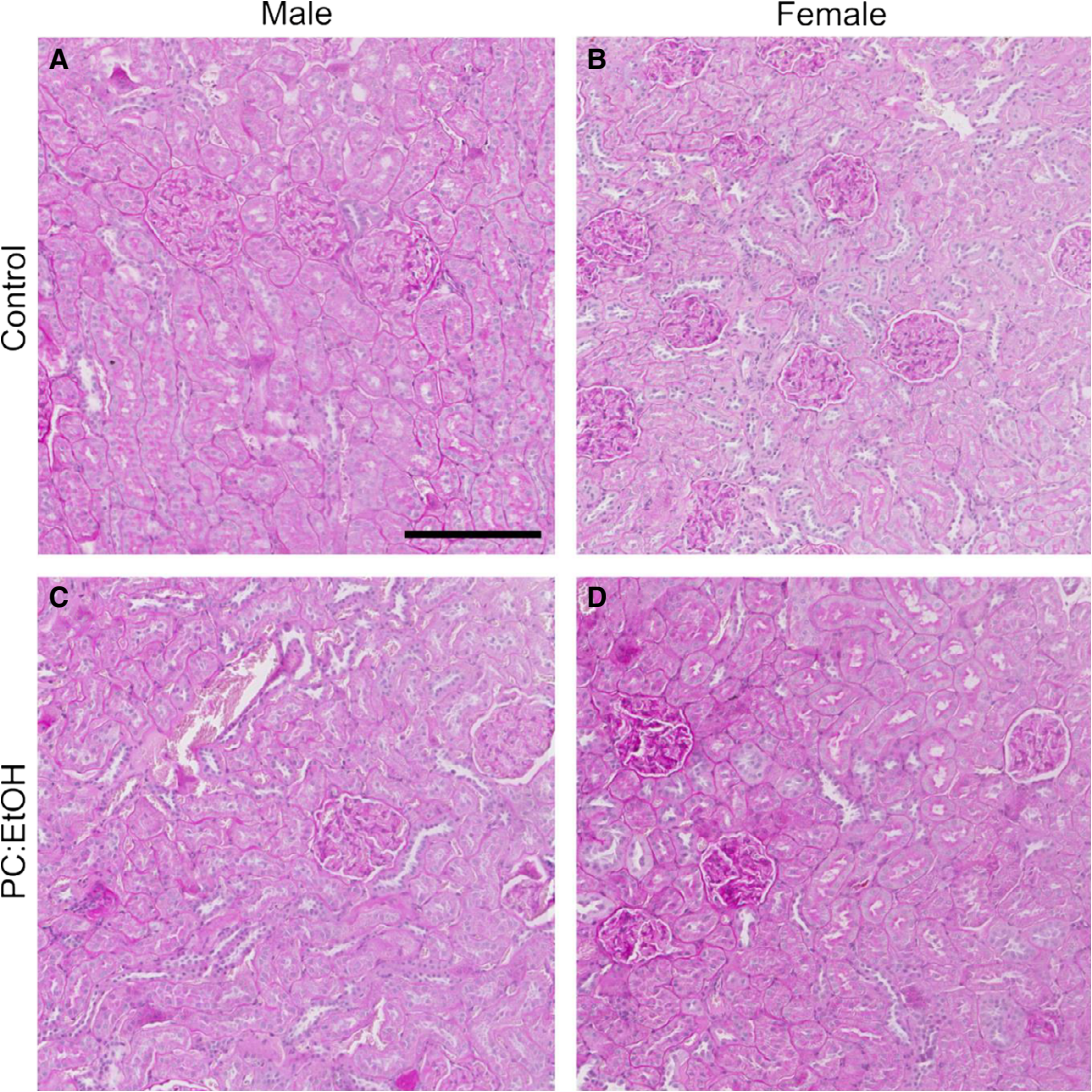
Representative PAS sections of offspring kidney medullary/cortex border from male (A,C) and female (B,D) rats at 19 months of age. Offspring were from dams treated with Control (A,B) or PC:EtOH diet (C,D). Scale bar is 200 *µ*m, *n* = 3 per group.

No differences between treatment groups were found upon histological examination of kidney sections from aged offspring of all groups. Representative PAS sections are shown Figure 5. Both control and PC:EtOH animals displayed rare eosinophilic casts (protein) in the cortical tubules, but without accompanying inflammation. There was some evidence of mild inflammation in one PC:EtOH female, but this was not representative of the group and likely an incidental finding.

### Renal gene expression and collecting duct length

**Figure 6:**
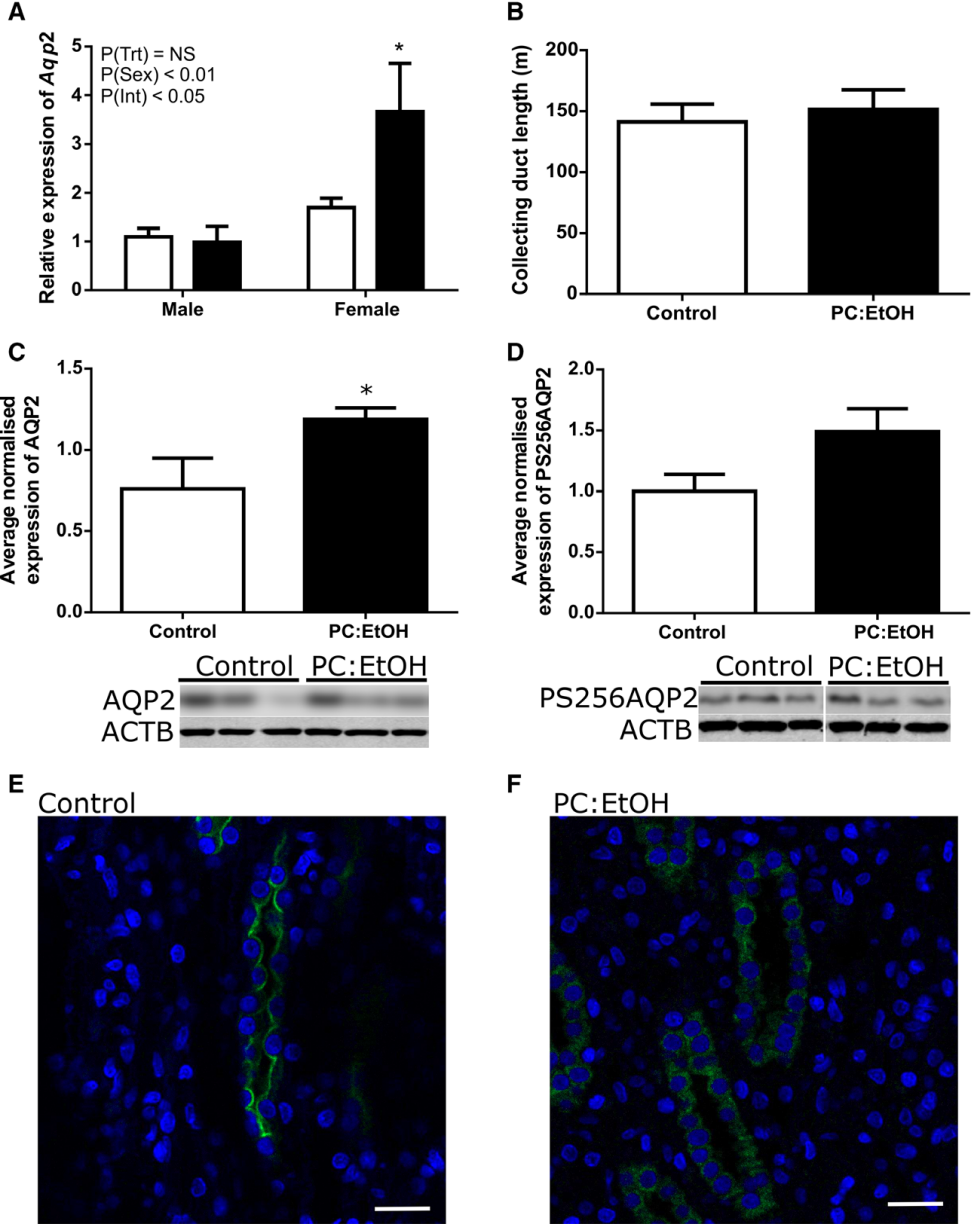
Relative expression of *Aqp2* mRNA (A, *n* = 6–10 per group), collecting duct length (B, *n* = 4) and normalized protein expression of AQP2 (C) and PS256AQP2 (D) in kidney lysates from female offspring from dams treated with control (White) or PC:EtOH diets (Black) (*n* = 6–7 per group). Representative images of immunofluorescent localization of AQP2 (green) and nuclear staining (Hoest, blue) in medullary kidney sections from female offspring of dams fed control (E) and PC:EtOH diets (F). In control animals, localization appeared concentrated on the apical membrane of the principal cells of the collecting duct, while in PC:EtOH females, staining was diffuse throughout the cytoplasm (*n* = 3). Data are mean ± SEM. Data are analysed using two‐way ANOVA with Bonferroni post‐hoc test (A) and Student's *t*‐test (B,C,D). *P < 0.05 when compared to same sex control. Western blot images are representative subsections of entire blots. For PS256AQP2, white line indicates break between bands. NS, not significant.

**Figure 7:**
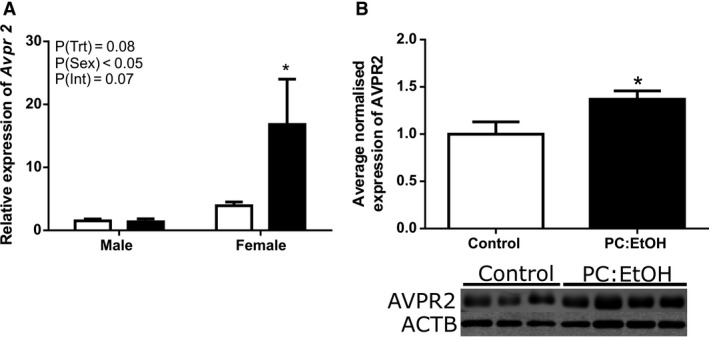
Relative expression of *Avpr2* mRNA (A, *n* = 6–10 per group) in kidneys from 19 month old offspring of dams treated with control (White) or PC:EtOH (Black) diet. Average normalized protein expression of AVPR2 in kidney lysates from kidneys from female offspring at 19 months of age (B, *n* = 6–7 per group). Data are mean ± SEM and are analysed using two‐way ANOVA (A) and Student's *t*‐test (B). *P < 0.05 when compared to control of same sex. Western blot images are representative subsections of entire blots.

*Aqp2* mRNA expression was significantly higher in females than in males (PSex < 0.01, Fig. 6A). An interaction between sex and treatment was found (PInt < 0.05) and post‐hoc analysis demonstrated that *Aqp2* expression was increased following PC:EtOH in the kidneys of female (*P* < 0.05), but not male offspring. Total collecting duct length did not differ between control and PC:EtOH females at 19 months of age (Fig. 6B), suggesting that the increase in *Aqp2* expression was not due to increased collecting duct surface area. *Avpr2* mRNA expression was higher in female kidneys than in males (PSex < 0.05, Fig. 7A) and post‐hoc analysis (*P* < 0.05) indicated that PC:EtOH female offspring had increased expression of *Avpr2* in kidneys when compared to control.

### Protein expression and localization

Using the average normalized expression from three Western blots using fresh protein, expression of non‐glycosylated AQP2 was increased in the kidneys of female offspring following PC:EtOH (*P* < 0.05, Fig. 6C). Phosphorylated AQP2 in the kidneys of female PC:EtOH offspring also increased (Fig. 6D), but to a lesser degree and this did not reach statistical significance. AVPR2 protein expression assessed by Western blot was increased in kidneys from aged female PC:EtOH offspring (*P* < 0.05, Fig. 7B).

Immunofluorescent labeling for AQP2 indicated altered distribution of AQP2 in the medullary collecting ducts within kidneys from female PC:EtOH offspring (Fig. 6E and 6F). In control female offspring, AQP2 immunoreactivity was localized to the apical membrane of principal cells of collecting ducts within both the renal medulla and the cortex. In PC:EtOH female offspring, AQP2 protein appeared diffusely distributed throughout the cell cytoplasm in the medullary collecting ducts. Localization of AQP2 in kidneys from male PC:EtOH offspring was similar to that of control males and control females (data not shown).

## Discussion

The major finding of this study was that periconceptional alcohol exposure reduced nephron endowment in male offspring and programmed long‐lasting alterations to urine flow, specifically, diuresis in female offspring. Intriguingly, this diuresis developed with age and occurred in a sexually dimorphic way. At 19 months of age, female offspring from the PC:EtOH group had greater urine flow but had increased expression of renal AQP2, a key mediator in free water reabsorption in the kidney. Furthermore, the expression of AVPR2, the receptor responsible for trafficking of AQP2, was also increased in females from PC:EtOH group only, but the phosphorylated AQP2 was not significantly different to control. These results suggest that the increased urine flow in females may be mediated by altered trafficking of AQP2 to the apical membrane of principal cells within the collecting duct. These findings are of clinical relevance due to the large proportion of women consuming alcohol prior to pregnancy recognition and the increased burden of health costs, particularly associated with chronic kidney disease.

We have shown previously that our model PC:EtOH exposure results in a peak blood alcohol concentration of ~0.14% 30 min after being offered the diet and thereafter, the BAC drops quickly, being 0.07% and 0.05% at 3 and 5 h respectively. Similar levels may be reached in women after consuming 4–5 standard drinks (40–50g of alcohol) over a couple of hours. Although alcohol was only given up until implantation, fetuses were mildly growth restricted in late gestation (Gardebjer et al. 2014). Here, we have shown that PC:EtOH offspring had largely caught up in weight by PN30, however, kidney weight was reduced compared to controls. Kidneys of male offspring exposed to PC:EtOH were smaller in proportion to body weight, indicating disrupted or slowed kidney development. This reduction in kidney weight was associated with a decrease in glomerular number of ~9% in males and ~6% in females. Decreased nephron number is common following prenatal perturbations, including uteroplacental insufficiency, prenatal ‘binge' alcohol exposure, and maternal exposure to elevated glucocorticoids (Wlodek et al. 2008; Gray et al. 2010; O'Sullivan et al. 2015). However, the deficit in this study was modest and less than observed with acute maternal alcohol exposure at mid gestation in the rat (15% and 10% in male and female offspring, respectively) (Gray et al. 2010) or prenatal glucocorticoids (dexamethasone: 25% decrease; corticosterone: 33% decrease in male mouse offspring) (O'Sullivan et al. 2015; 2013). Of note, the majority of animal studies reporting alterations to nephron number examined perturbations that occur during the critical period of nephrogenesis. Here we have reported a reduction in nephron number despite alcohol exposure occurring some 10 days prior to the start of development of the permanent kidney. This suggests that alcohol may act indirectly, perhaps via placental insufficiency, to slow growth and development of the kidney. Placental insufficiency has been shown to reduce nephron endowment in offspring (Moritz et al. 2009) and alcohol exposure *in vitro* has been shown to reduce trophoblast survival (Kalisch‐Smith et al. 2016). We have recently demonstrated that placental development is impaired in our PC:EtOH model possibly due to epigenetic changes in the early blastocyst (Kalisch‐Smith et al. 2019). Furthermore, PC:EtOH exposure is associated with alterations in placental glucose transporter expression and glycogen accumulation at late gestation, highlighting impairments in placental function and/or structure in our model (Gardebjer et al. 2014).

The “Brenner hypothesis” links a decrease in nephron endowment to glomerular hyperfiltration, resulting in glomerular damage, elevation of arterial blood pressure, and deficits in sodium and fluid homeostasis (Brenner et al. 1988). A previous study of mid gestation alcohol exposure in rats reported sex‐specific alterations in glomerular filtration rate and increased mean arterial pressure in offspring at 6 months of age (Gray et al. 2010). Other prenatal perturbations, such as maternal glucocorticoid exposure, have been associated with a low nephron endowment and increases in blood pressure (Singh et al. 2010; O'Sullivan et al. 2013). Despite observing a decreased nephron endowment following PC:EtOH, we did not observe alterations in the blood pressure profiles of offspring when measured by radiotelemetry. This finding was perhaps not surprising as the nephron deficit was less than other models linking nephron number and blood pressure. In addition, we have recently reported that exposure to a low dose of alcohol throughout pregnancy does not increase blood pressure in rat offspring (Walton et al. 2019) although it does cause changes in cardiac function (Nguyen et al. 2014 ). We speculate that a secondary lifestyle insult such as a high salt or high fat diet may be required to induce overt signs of disease following PC:EtOH. Salt sensitive hypertension has been demonstrated during a high sodium challenge in rats following a prenatal low protein diet (Woods et al. 2004), and in our model of PC:EtOH, we have recently demonstrated that a Western style diet exacerbates the metabolic outcomes, including the development of obesity in male offspring (Gardebjer et al. 2015; 2018). This highlights the ability of postnatal insults to exacerbate underlying conditions following prenatal perturbations.

At 6 months of age we observed no alterations to urine flow or electrolyte/protein excretion either during hydrated or dehydrated conditions in animals exposed to PC:EtOH. This was surprising given the literature linking prenatal perturbations, including alcohol, to early life renal deficits. A low protein diet throughout pregnancy has been shown to cause increases in urine production, salt excretion, and water intake at 60 days of age in rodents (Alwasel et al. 2012). Prenatal alcohol exposure (35% ethanol derived calories from day 6 of gestation) also induced growth restriction at birth and, by 3 months of age, offspring had increased fractional sodium excretion and decreased fractional potassium excretion, a phenotype that was exacerbated during sodium restriction (Assadi et al. 1991). A late gestation binge‐like model of alcohol exposure (2.0 g/kg) has been shown to decrease basal sodium and potassium concentrations in urine at PN30 as well as cause alterations to AVP cells in the paraventricular nucleus of offspring (Godino et al. 2015).

Whilst our results suggest that PC:EtOH does not cause renal dysfunction at an early age, there is evidence that renal function, including urinary concentrating ability, worsens with age (Combet et al. 2001; Singh et al. 2011). Impairments in the ability to concentrate urine have been shown to be an early marker of renal disease in children (Garcia Nieto et al. 2008). This led us to repeat the renal function studies in the same animals at an advanced age. At 19 months of age, urine flow in females exposed to PC:EtOH was higher, and this persisted during a 24 h dehydration challenge. Similar results have been reported in 3‐month old offspring following prenatal alcohol exposure (35% ethanol derived calories) from days 7–22 of pregnancy (Knee et al. 2004). The authors attributed the increased urine flow to decreased expression of *Avp* mRNA in the hypothalamus but found no alteration to circulating basal plasma AVP levels, indicating a partial diabetes insipidus phenotype. Using this model, prenatally alcohol‐exposed rats had a reduced AVP response to hemorrhage suggesting changes in regulation of AVP secretion under volume stress. This was associated with decreased hypothalamic and pituitary AVP content, with no changes to blood pressure (Bird et al. 2006). In our model, the PC:EtOH female offspring did drink more water suggestive of changes in thirst mechanisms. However, PC:EtOH did not alter plasma AVP levels, either basally or during a dehydration (osmotic) challenge, suggesting inappropriate AVP production and/or release is not the cause of the increased urine flow in PC:EtOH females. Further investigation into other mechanisms regulating thirst is warranted in the female offspring exposed to PC:EtOH.

We next considered renal mechanisms that may contribute to the diuresis observed in PC:EtOH female offspring. One potential explanation for the increased urine flow may be altered structure of the renal medulla, which contains the collecting ducts that are the final site of water reabsorption in the kidney. We used a novel method of unbiased stereology (Walton et al. 2016) to show total collecting duct length in kidneys of female offspring did not differ between control and PC:EtOH at either PN30 or 19 months of age. This suggests that the increase in urine flow in female PC:EtOH offspring was not the result of deficits in collecting duct elongation that may have occurred in conjunction with the deficit of nephrons. Water consumption was increased following PC:EtOH in female offspring during 24hr metabolic cage studies. Similarly we have reported increased water intake, measured over 4 days in home cages, in females of the same cohort of animals at 12 months of age (Dorey et al. 2018). An increase in water intake and urine flow has been reported for a rat model of diabetes mellitus, and this was associated with changes in inner‐medullary AQP2, phosphorylated AQP2 and AQP3 expression (Nejsum et al. 2001). Water homeostasis and free water movement into the urine filtrate is a tightly regulated process in which AQP2 abundance in the apical membrane is the rate limiting step. Following AVP binding to the V_2_ receptor on principal cells of the collecting duct, phosphorylation of vesicle‐held AQP2 allows an increase in water transport (for review, see (Moeller and Fenton, 2012)). Given the paradoxical increase in both basal mRNA and protein expression of AVPR2 despite the increased urine flow, we explored whether phosphorylation and trafficking of AQP2 was occurring appropriately upon binding of AVP to AVPR2. The activity of AQP2 is dependent upon its phosphorylation and trafficking to the cell membrane (Gooch et al. 2006). Using qualitative AQP2 immunofluorescence, we demonstrated AQP2 was more diffusely expressed within the cytoplasm and less localized to the apical membrane of principal cells. Further to this, although there was a tendency for phosphorylated AQP2 (PS256AQP2) to be increased by PC:EtOH, this did not reach significance. This phosphorylation event has been shown to be essential for membrane accumulation of AQP2 (Arthur et al. 2015). Furthermore, urinary cAMP levels, which can be used as a marker of AQP2 phosphorylation were not different following PC:EtOH. Our results suggest that while increases in AQP2 protein expression occurred, inappropriate phosphorylation of AQP2 resulted in impaired trafficking to the apical membrane in the collecting duct, thereby preventing appropriate water reabsorption.

Finally, we demonstrated that not only does urine flow increase with age following PC:EtOH, but that it does so in a sex‐specific manner. Females ordinarily have higher levels of AVPR2 in the kidney compared to males (Liu et al. 2011). Ovariectomized female rats displayed higher phosphorylated‐AQP2 in their kidneys, and *in vitro* studies have shown that direct estrogen stimulation of the mpkCCD principal cell line decreased renal AQP2 mRNA and protein (Cheema et al. 2015). Interestingly expression of ERα was shown to be important in mediating the inhibitory effect of oestradiol on AQP2 expression. These studies suggest that whilst circulating AVP levels were unchanged following PC:EtOH, either the renin‐aldosterone‐system or circulating estrogen may be affected. This warrants investigation in future studies.

In conclusion, our study is the first to show that alcohol consumption, limited to the time around conception, adversely affects renal development as shown by reduced glomerular number in both male and female offspring. Although urine flow was unaffected at 6 months of age, aged female offspring exposed to periconceptional alcohol had increased urine flow and aberrant localization of the AQP2 protein within the medullary collecting ducts under basal conditions. In contrast, these alterations in urine flow and AQP2 expression were not observed in male offspring. This adds to the growing body of evidence that perturbations around the time of conception can affect development even prior to organogenesis. These results highlight the importance of avoiding alcohol when planning a pregnancy due to potential long‐term health consequences for offspring.

## Conflict of Interest

The authors have no conflict of interest to declare.
